# Value of plasma metagenomic next-generation sequencing for the diagnosis of invasive aspergillosis: a multicenter-center retrospective study

**DOI:** 10.3389/fcimb.2025.1656233

**Published:** 2025-11-21

**Authors:** Xiaomai Wu, Wei Wang, Liang Xu, Wei Zhou, Jianying Zhou, Hua Zhou

**Affiliations:** 1 Department of Respiratory and Critical Care Medicine, The First Affiliated Hospital, Zhejiang University School of Medicine, Hangzhou, China; 2 Department of Respiratory and Critical Care Medicine, Taizhou Hospital, Taizhou, China; 3 Department of Respiratory and Critical Care Medicine, The Affiliated Hospital, Shaoxing University, Shaoxing, China; 4 Department of Respiratory and Critical Care Medicine, Shaoxing Central Hospital, Shaoxing, China

**Keywords:** plasma, MNGs, aspergillus, invasive aspergillosis, microbial cell free-DNA

## Abstract

**Introduction:**

Invasive aspergillosis (IA) is a severe fungal infection. Metagenomic Next Generation Sequencing (mNGS) is abroad and highly sensitive pathogen detection method that can accurately differentiate fungi to the species, and even subspecies level.

**Methods:**

To explore the value of plasma mNGSs in the diagnosis of invasive aspergillosis, a retrospective analysis was conducted on the clinical data of 334 patients with findings of *Aspergillus* spp. From mNGS from plasma at 4 hospitals, Zhejiang, from February 2021 to December 2022. The study analyzed risk factors, clinical manifestations, imaging features, microbiological results, and treatment outcomes of patients with *Aspergillus* infection.

**Results and discussion:**

According to the diagnostic criteria for IA, among the 334 patients, there were 4 confirmed cases, 62 probable cases, 134 possible cases, and 134 false-positive cases. All 196 probable and possible cases exhibited risk factors, clinical manifestations, imaging features, and treatment outcomes consistent with *Aspergillus* infection. In 18 out of the 62 probable cases, the same *Aspergillus* nucleic acid was found in 2–4 peripheral blood mNGS samples collected at intervals of 17 days. The remaining 134 patients had detectable *Aspergillus* in plasma mNGS but lacked high-risk factors and clinical characteristics of *Aspergillus* infection, and there was a lack of other microbiological evidence, determined as false positives. Among the cases included in this study, the positive predictive value of plasma mNGS for diagnosing invasive aspergillosis was 59.9%. Plasma mNGS detection has significant reference value for diagnosing IA. However, comprehensive judgment should still be made in conjunction with clinical features.

## 1 Introduction

Invasive aspergillosis (IA) is a severe fungal infection caused by *Aspergillus*, often occurring in patients with severe immunodeficiency, such as those with hematological malignancies, hematopoietic stem cell transplantation, solid organ transplantation, etc. Mortality is high in patients who do not receive early and appropriate antifungal treatment (Yerbanga et al., 2023). There is an increasing number of reports of IA in critically ill patients in the intensive care unit, especially in those with risk factors such as corticosteroid use, chronic obstructive pulmonary disease, liver failure, malnutrition, chronic kidney disease, diabetes, severe viral pneumonia (such as Influenza, COVID-19), etc (Bassetti et al., 2021).

Timely and accurate diagnosis with effective treatment is a key factor in improving prognosis and reducing unreasonable use of antibiotics. The definitive diagnosis of *Aspergillus* infection relies on microscopic examination or culture. Microbiological evidence for the clinical diagnosis of aspergillosis mainly includes microscopic examination, cultures, Aspergillus galactomannan, or nucleic acid detection. However, the positive rate of culture for clinical samples is low. In 2020, *Aspergillus* PCR (Molecular Diagnostic Assays) was included in the definition of invasive aspergillosis, requiring two positive results to provide sufficient specificity for confirmation of the diagnosis (Koehler et al., 2021). Metagenomic Next Generation Sequencing (mNGS) is a broad and highly sensitive pathogen detection method that can accurately differentiate fungi to the species, and even subspecies level. This technology has the potential to improve the sensitivity of detection for invasive fungal infections (Jenks et al., 2023). Therefore, we aim to explore the diagnostic value of plasma mNGS in *Aspergillus* infections. Here, we retrospectively analyzed the clinical data of 167 patients with detectable *Aspergillus* spp. in plasma mNGS at 4 hospitals, Zhejiang, from February 2021 to December 2022, and the findings are summarized as follows.

## 2 Objectives and methods

### 2.1 Study subjects

A total of 4705 patients underwent peripheral blood mNGS testing at 4 hospitals, Zhejiang, between 01/02/2021 and 30/12/2022. Patients who were found to have detectable nucleic acid of the *Aspergillus* genus were included. Clinical comprehensive diagnoses of invasive aspergillosis or non-infection were made by three senior experts in respiratory medicine, infectious diseases, and radiology. This decision was reached through discussions with the patient’s primary medical team, considering clinical symptoms, laboratory results, imaging studies, microbiology, pathology examinations, as well as treatment, prognosis, etc. Cases with incomplete clinical data, individuals under 18 years old, pregnant or postpartum women, and cases of non-hospitalized patients were excluded.

### 2.2 Relevant definitions

IA is categorized into confirmed, probable and possible diagnosis. Cases included in this study had at least one instance of blood mNGS detecting *Aspergillus*, upon which the diagnosis was based. The diagnostic criteria followed the guidelines for invasive fungal diseases jointly published by the European Organization for Research on Treatment of Cancer (EORTC) and the Mycoses Study Group Education and Research Consortium (MSGERC) in 2020 (Pappas et al., 2021). Patients were categorized into confirmed, probable, and possible diagnoses of IA based on these diagnostic criteria.

Confirmed Diagnosis: Criteria: Pathological findings from surgical or biopsy tissue at the infection site confirm *Aspergillus* infection.

Probable Diagnosis: Host factors, clinical symptoms or signs, and microbiological criteria consistent with *Aspergillus* infection are required. The microbiological criteria for clinical diagnosis are as follows: *Aspergillus* identified by smear or culture from specimens at the infection site, and detection of the same *Aspergillus* species in at least two mNGS tests (including blood or specimens from the infection site).

Possible Diagnosis: Host factors and clinical features consistent with *Aspergillus* infection. Host factors include hematological malignancies, solid organ and hematopoietic stem cell transplantation (HSCT), chemotherapy or immunotherapy for solid tumors, granulocyte deficiency, prolonged use of corticosteroids (average minimum dose exceeding 0.3 mg/kg/day for more than 3 weeks of prednisone or equivalent), ICU critically ill patients, chronic obstructive pulmonary disease, diabetes, liver failure, renal failure, etc (Gaffney et al., 2023).

### 2.3 Testing for *Aspergillus* spp.

The diagnostic methods for *Aspergillus* spp. include fungal culture, calcofluor white stain for fungi, galactomannan (GM) antigen test. The GM antigen was detected using a Platelia Aspergillus EIA kit (Bio-Rad, Marnes-la-Coquette, France), according to the manufacturer’s instructions. Both positive and negative controls were included in each assay. A result with an index value of >0.5 (serum) or 0.8 (BALF) in duplicate tests was considered positive.

### 2.4 Plasma mNGS testing

The testing protocol, experimental parameters, quality control, and test result reporting of the plasma mNGS testing in our laboratory are described by Han et al. (7) Briefly, peripheral venous blood samples were collected and sent to the clinical laboratory as soon as possible. Plasma was separated under the centrifugal parameters of 500 g for 5 min at 4°C. The separated plasma is used to extract cell free DNA (cfDNA) and cell free RNA (cfRNA) and libraries were constructed. The size distribution was measured and the concentration of the libraries was quantified. Library pools were then loaded onto the Illumina Nextseq CN500 sequencer for 50 cycles of single-end sequencing (SE-50), generating approximately 20 million reads for each library. Microbial reads were aligned to the database using Burrows-Wheeler Aligner software which contains a proprietary curated database consisting of more than 20,000 microbial reference genomes. Most mNGS results returned within 36 hours.

### 2.5 Data collection

Demographic information, risk factors, underlying diseases, use of immunosuppressive drugs, steroids, chemotherapy drugs in the month preceding onset, clinical symptoms, laboratory examinations, imaging data, as well as treatment and prognosis, etc., were collected for the 334 included patients.

### 2.6 Comparison of clinical characteristics between IA and contaminated patients

Statistical comparisons were made between patients with positive blood mNGS for IA and contaminated patients. General conditions, risk factors, clinical manifestations, and prognosis were compared.

### 2.7 Data processing

SPSS 27.0 software (IBM Corporation, Chicago, Illinois, USA) was used for data analysis. Continuous variables conforming to a normal distribution were expressed as mean ± standard deviation (x ± s), and an independent sample t-test was used for intergroup comparison of measurement data. Non-normally distributed continuous variables were expressed as median and interquartile range, and the Mann-Whitney U test was used for comparison. Count data were compared using the chi-square test or Fisher’s exact test. A P value < 0.05 indicated a statistically significant difference.

## 3 Results

### 3.1 General characteristics

**Figure 1:**
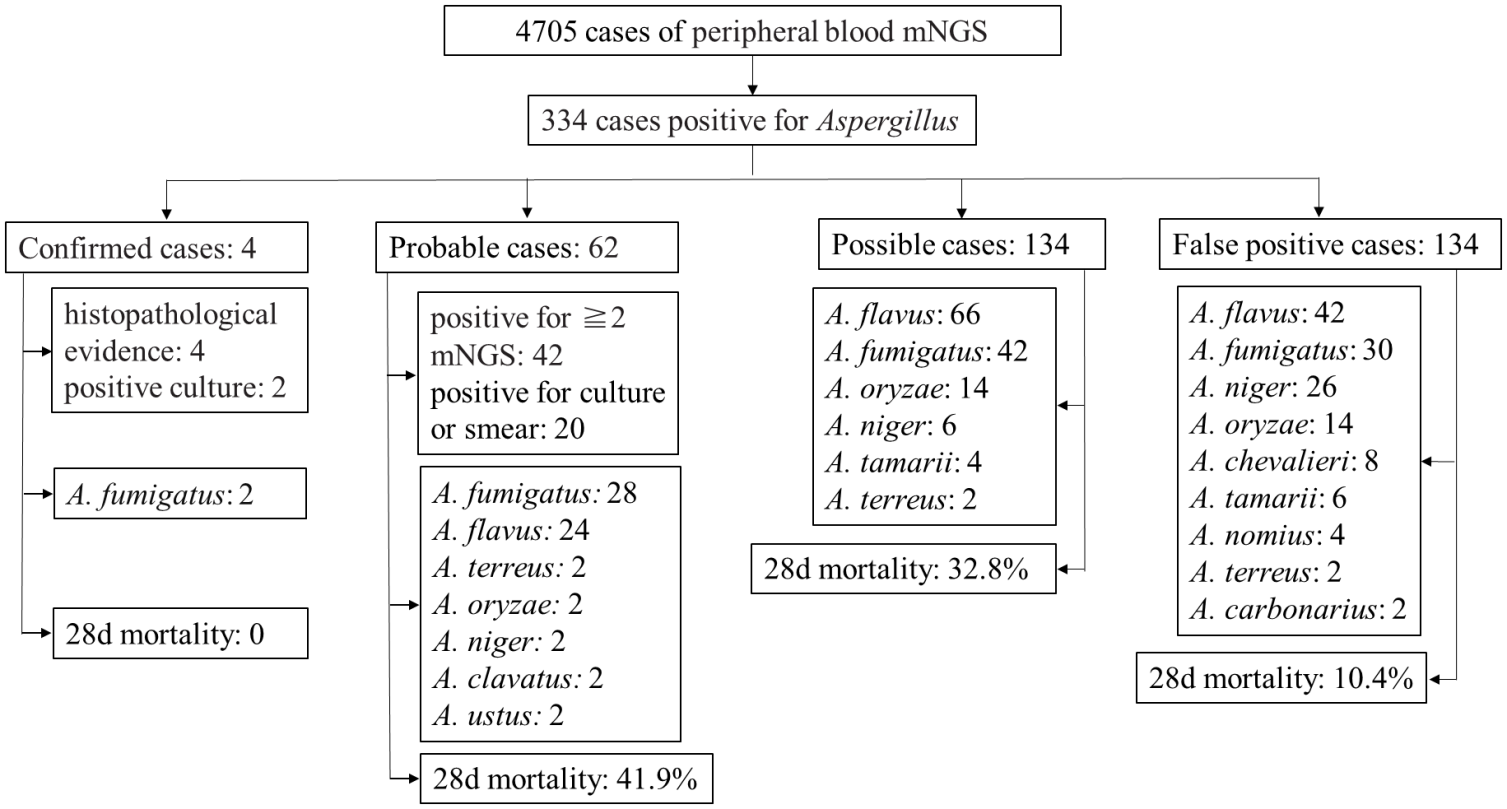
Overall clinical and pathogen diagnosis of the recruited patients.

A total of 334 patients were included, comprising 188 males (56.3%) and 146 females (43.7%), with a median age of 58 years (range 43 to 68). Underlying diseases included hematological malignancies in 202 cases, with 128 cases of leukemia (38.3%), 34 cases of lymphoma (10.2%), 26 cases of myelodysplastic syndrome (7.8%), and 14 cases of multiple myeloma (4.2%). There were 54 cases post-hematopoietic stem cell transplantation (16.2%), 36 cases post-solid organ transplantation (10.8%), and 44 cases of diabetes (13.2%). Other underlying conditions included 32 cases of solid tumors (9.6%), among others, **Table 1**. Overall clinical and pathogen diagnosis of the recruited patients are showed in **Figure 1**.

**Table 1:** Clinical characteristics of enrolled patients.

Clinical Man	Overall (n=334)
Age (range)	58 (43 to 68)
Sex	
Male, n (%)	188 (65.3)
Female, n (%)	146 (43.7)
Symptoms, n (%)	
Fever	292 (87.4)
Cough	276 (82.6)
Purulent Sputum	154 (46.1)
Hemoptysis	38 (11.3)
Chest Pain	34 (10.2)
Dyspnea	196 (58.7)
Underlying diseases, n (%)	
Leukemia	128 (38.3%)
Lymphoma	34 (10.2)
Myelodysplastic syndrome	26 (7.8)
Multiple myeloma	14 (4.2)
Hematopoietic stem cell transplantation	54 (16.2)
Solid organ transplantation	36 (10.8)
Diabetes	44 (13.2)
Solid tumors	32 (9.6)
Antibiotic Exposure, n (%)	334 (100)

### 3.2 Confirmed cases

There were 4 patients with confirmed invasive pulmonary aspergillosis based on histopathological evidence. There were 2 men and 2 women, 2 patients with lung transplantation, 2 patients with hematological malignancies, 1 patient with diabetes and 1 patient with bronchiectasis. Four patients were further confirmed to have *Aspergillus* infection through bronchoscopy biopsy and histopathological examination. In addition, both patients were confirmed to have *Aspergillus* infection in bronchoalveolar lavage fluid culture and mNGS testing. The *Aspergillus* infection in all 4 patients improved after treatment.

### 3.3 Probable cases

There were 62 cases, including 32males, aged 21-95 (56.1 ± 16.7) years. Among them, 58 cases were clinically diagnosed with invasive pulmonary aspergillosis, 4 case with disseminated invasive aspergillosis, and 4 case with urinary system *Aspergillus* infection. Underlying diseases included 44 cases of leukemia, 4 cases of lymphoma, 4 cases of myelodysplastic syndrome, 2 case of multiple myeloma, 10 cases after solid organ transplantation, 12 cases after hematopoietic stem cell transplantation, 4 cases of diabetes, 2 case of chronic obstructive pulmonary disease, and 6 cases of solid tumors. Nine patients had the same genus *Aspergillus* detected in 2–4 peripheral blood mNGS tests with an interval of 16 (10, 61) days. 20 cases were positive in culture and smear. 24 cases had the same genus of *Aspergillus* detected by using mNGS in sputum, lavage fluid, or cerebrospinal fluid. Among the 62 cases, there were 28 cases of *A. fumigatus*, 24 cases of *A. flavus*, 2 case of *A. terreus*, 2 case of *A. oryzae*, 2 case of *A. niger*, 2 case of *A. clavatus*, and 2 case of *A. ustus*. Pulmonary CT of 58 IPA patients showed imaging features consistent with *Aspergillus* infection, including nodular shadows, patchy shadows, halo signs, cavities, consolidation, etc. After active treatment, 36 cases improved, and 26 cases died, 28d mortality: 41.9%.

### 3.4 Possible cases

There were 134 suspected cases, including 84 males (62.7%). These patients, in addition to one instance of blood mNGS detecting *Aspergillus*, also presented with both the risk factors for *Aspergillus* infection and clinical features consistent with *Aspergillus* infection. Clinical characteristics are detailed in **Table 2**.

**Table 2:** Clinical characteristics of 134 patients with possible invasive aspergillosis.

Clinical characteristics (n=134)		N (%)
Demographic characteristics	Male	84 (62.7%)
	Female	50 (37.3%)
	Age (year)	124 (46, 72)
Risk Factors	Leukemia	66 (49.3%)
	Hematopoietic Stem Cell Transplantation	20 (14.9%)
	Myelodysplastic Syndrome	10 (7.5%)
	Lymphoma	12 (9.0%)
	Multiple Myeloma	10 (7.5%)
	Solid Organ Transplantation	8 (6.0%)
	Diabetes Mellitus	20 (14.9%)
	ICU Admission	24 (17.9%)
	Liver Failure	6 (4.5%)
	Chronic Obstructive Pulmonary Disease	6 (4.5%)
	Solid Tumors	6 (4.5%)
	Corticosteroids	2 (1.5%)
Radiological Findings in Cases of Pulmonary Infection	Nodule	52 (38.8%)
	Mass-like Shadow	26 (19.4%)
	Patchy Shadow	46 (34.3%)
	Reversed Halo Sign	2 (1.5%)
	Consolidation	16 (11.9%)
	Cavity	8 (6.0%)
	Pleural Effusion	6 (4.5%)
	Halo Sign	2 (1.5%)
	Crescent Sign	2 (1.5%)
	Infiltration	2 (1.5%)
	Tree-in-Bud Sign	4 (3.0%)
Infection site	Lungs	132 (98.5%)
	Disseminated infection	2 (1.5%)
Aspergillus genus	*A. flavus*	66 (49.3%)
	*A. fumigatus*	42 (31.3%)
	*A. oryzae*	14 (10.4%)
	*A. niger*	6 (4.5%)
	*A. tamarii*	4 (3.0%)
	*A. terreus*	2 (1.5)
Mortality	14-day mortality	40 (29.9%)
	28-day mortality	44 (32.8%)

### 3.5 Contaminated cases

There were 134 contaminated cases, including 70 males (52.2%). The age ranged from 18 to 90 years (mean ± SD, 51.3 ± 17.9). Clinical characteristics are detailed in **Table 3**.

**Table 3:** Clinical characteristics of 134 false-positive cases.

Clinical characteristics (n=134)		N (%)
Demographic characteristics	Male	70 (52.2%)
	Female	64 (47.8%)
	Age (years)	51.3 ± 17.9
Risk factors	Leukemia	40 (29.9%)
	Lymphoma	18 (13.4%)
	Myelodysplastic Syndrome	12 (9.0%)
	Multiple Myeloma	2 (1.5%)
	Solid Tumors	18 (13.4%)
	Kidney Transplantation	8 (6.0%)
	Diabetes Mellitus	18 (13.4%)
	Hematopoietic Stem Cell Transplantation	22 (16.4%)
	Corticosteroids	14 (10.4%)
	ICU admission	12 (9.0%)
	Liver Failure	2 (1.5%)
*Aspergillus* genus identified by mNGS	*A. flavus*	42 (31.3%)
	*A. fumigatus*	30 (22.4%)
	*A. niger*	26 (19.4%)
	*A. oryzae*	14 (10.4%)
	*A. chevalieri*	8 (6.0%)
	*A. tamarii*	6 (4.5%)
	*A. nomius*	4 (3.0%)
	*A. terreus*	2 (1.5%)
	*A. carbonarius*	1 (1.5%)
Mortality	14-day mortality	10 (7.5%)
	28-day mortality	14 (10.4%)

### 3.6 Other microorganisms except *Aspergillus* spp. detected in plasma mNGS of 167 patients

Only *Aspergillus* was detected in the plasma of 84 patients, and other microorganisms were detected in the plasma of 250 patients. The combined detected microorganisms are listed in **Table 4**. In IA group and false positive group, there was no significant difference in the detection of pathogens other than *Aspergillus* spp.

**Table 4:** Other microorganisms except *Aspergillus* spp. detected in plasma mNGS of 250 patients.

Microorganisms	Total (n=250)	IA group (n=146)	False positive group (n=106)
Herpesvirus type 1	44	28	16
Herpesvirus type 4	62	42	20
Herpesvirus type 5	106	70	36
Herpesvirus type 7	2	0	2
Herpesvirus type 8	2	0	2
Human parvovirus B19	10	6	4
WU Polyomavirus	2	2	0
Torque teno virus	10	10	0
Hepatitis B virus	12	10	2
GB virus C/hepatitis G virus	46	34	12
New Bunia virus	4	2	2
SARS-Cov-2	2	2	0
Influenza A H3N2	2	0	2
Candida albicans	14	10	4
Candida tropicalis	10	6	4
Pneumocystis jiroveci	8	8	0
Cunninghamella sp	6	4	2
Rhizopus sp	2	2	0
Lichtheimia sp	2	0	2
*Staphylococcus aureus*	8	8	0
Staphylococcus epidermidis	2	0	2
Corynebacterium striatum	6	6	0
Enterococcus faecium	16	10	6
Enterococcus faecalis	6	0	6
Streptococcus angina	2	2	0
Nocardia melitensis	2	0	2
Acinetobacter baumannii	8	8	0
Pseudomonas aeruginosa	16	8	8
Klebsiella pneumoniae	22	12	10
Escherichia coli	8	4	4
Enterobacter asheri	2	2	0
Burkholderia cepacia	2	2	0
Haemophilus parahaemolyticus	2	0	2
Bacteroides fragilis	2	0	2
Veillonella atypica	2	0	2
Ureaplasma urealyticum	2	2	0
Cryptococcus neoformans	4	2	2
Mycobacterium tuberculosis	4	0	4

### 3.7 Comparison of clinical characteristics between patients with invasive aspergillosis and contaminated cases

A comparison of clinical data between 200 cases of blood mNGS-positive invasive aspergillosis and 134 contaminated cases revealed that the infection group had a higher incidence of lung imaging consolidation, masses, and cavities. There was no statistically significant difference in the read counts of Aspergillus between the infection group and the false positive group. Patients in the infection group had lower platelet counts, higher CRP levels, more critical cases, and poorer prognosis. **Table 5**.

**Table 5:** Comparison of clinical characteristics between the *Aspergillus* infection and false positive group.

Clinical Characteristics		Total (n=334)	Infection (n=200)	False positive (n=134)	P value
Demographic characteristics	Male	188 (56.3%)	118 (59%)	70 (52.2%)	0.388
	Female	146 (43.7%)	82 (41%)	64 (47.8%)	
	Age (year)	58 (43,68)	57.81 ± 15.68	51.34 ± 17.88	0.022*
Risk factors	Hematologic Malignancies	202 (60.5%)	130 (65%)	72 (53.7%)	0.144
	Hematopoietic Stem Cell Transplantation	54 (16.2%)	32 (16%)	22 (16.4%)	0.943
	Solid Organ Transplantation	36 (10.8%)	22 (11%)	8 (6.0%)	<0.001*
	Solid Tumors	32 (9.6%)	14 (7%)	18 (13.4%)	0.166
	Diabetes	44 (13.2%)	26 (13%)	18 (13.4%)	0.935
	Glucocorticoids	24 (7.2%)	10 (5%)	14 (10.4%)	0.303
	ICU Admission	58 (17.4%)	46 (23%)	12 (9%)	0.019*
	Liver Failure	20 (6%)	18 (9%)	2 (1.5%)	0.095
Radiological Findings in Cases of Pulmonary Infection	Reversed Halo Sign	4 (1.2%)	4 (2%)	0	0.517
	Halo Sign	6 (1.8%)	6 (3%)	0	0.403
	Nodule	102 (30.5%)	70 (35%)	32 (23.9%)	0.126
	Patchy Shadow	96 (28.7%)	68 (34%)	28 (20.9%)	0.067
	Mass-like Shadow	38 (11.4%)	38 (19%)	0	<0.001*
	Pleural Effusion	26 (7.8%)	14 (7%)	12 (9%)	0.644
	Infiltration	6 (1.8%)	6 (3%)	0	0.403
	Consolidation	28 (8.4%)	28 (14%)	0	0.001*
	Ground-glass opacity	16 (4.8%)	2 (1%)	14 (10.4%)	0.015*
	Cavity	20 (6%)	20 (10%)	0	0.019*
	Crescent Sign	4 (1.2%)	4 (2%)	0	0.517
Laboratory tests	WBC (×10^9/L)	1.75 (0.21,6.43)	1.16 (0.17, 5.54)	3.28 (0.36,7.63)	0.073
	NE (×10^9/L)	1.06 (0.03,4.9)	0.49 (0.36, 7.63)	1.96 (0.07, 5.94)	0.062
	PLT (×10^9/L)	36.5 (13,121)	24 (11, 65.25)	84.5 (17, 176.75)	<0.001*
	ALD (g/L)	33.09 ± 4.54	32.66 ± 4.48	33.73 ± 4.58	0.124

## 4 Discussion

*Aspergillus*, a ubiquitous saprophytic fungal pathogen, comprises over 250 species (Han et al., 2023), with *A. fumigatus* and *A. flavus* being the most common. *Aspergillus* produces abundant conidia, with each conidiophore generating thousands of conidia. These conidia, characterized by a small diameter (2 to 3 μm), can reach the alveoli when released into the atmosphere (Tegelaar and Wösten, 2017; Latgé and Chamilos, 2019). Environmental surveys indicate that humans inhale hundreds of *Aspergillus* conidia daily (Tanaka et al., 2015). Consequently, the lungs are the most common site for *Aspergillus* infections, with invasive pulmonary aspergillosis accounting for 98% of the 100 cases of *Aspergillus* infection in this study.

Due to the invasiveness and high mortality of *Aspergillus* infections, it is crucial for clinicians to rapidly and efficiently identify *Aspergillus* and initiate effective treatment. Microscopic examination and culture form the basis for diagnosing *Aspergillus* infections but lack sensitivity. Blood cultures have a low positivity rate and are prone to contamination, limiting their diagnostic value. Histopathological examination relies on invasive methods to obtain infected tissue, often constrained by critically ill patients, low platelet counts, or coagulation disorders. Currently, our challenge lies in the absence of non-invasive, rapid, and reliable diagnostic methods.

With the advent of more powerful methods such as novel PCR testing, mNGS, nanotechnology-based tools, and artificial intelligence-based models, the landscape of fungal diagnosis is continuously evolving in a positive direction. Molecular diagnostic methods, being rapid, effective, and non-invasive, allow for convenient sampling from blood, urine, sputum, and other specimens. The International Fungal PCR Initiative is actively working to incorporate fungal PCR diagnostics into the European Organization for Research on Treatment of Cancer (EORTC) and the Mycoses Study Group Education and Research Consortium (MSG) guidelines. Notably, *Aspergillus* PCR diagnostics have been successfully integrated into the EORTC/MSG guidelines for the diagnosis of aspergillosis. Aspergillus PCR exhibits high sensitivity, reaching up to 96% in routine blood samples, with excellent specificity, often reaching 100% in certain studies (Herbrecht et al., 2005). mNGS allows unbiased detection of various pathogens, including *Aspergillus*, with a short detection period. It enables identification at the species level and is less influenced by prior antifungal therapy (Cornely et al., 2014; Millon et al., 2016). For challenging severe cases suspected of *Aspergillus* infection, specimens such as blood and bronchoalveolar lavage fluid can be subjected to mNGS testing (Cornely et al., 2019). This study found that the positive predictive value of blood mNGS in diagnosing invasive aspergillosis was 59.9%, emphasizing the need for careful result interpretation and the importance of combining clinical features while actively seeking additional evidence of *Aspergillus* infection.

Our retrospective study failed to answer the sensitivity of plasma mNGS in the diagnosis of IA. According to the multiplex PCR detection results of *Aspergillus* reported in the literature (Herbrecht et al., 2005), the specificity of multiplex PCR in the diagnosis of IA is better than that of mNGS in this study. Multiplex PCR will be cheaper and shorter detection time, which has certain advantages. However, most of the patients enrolled in this study are immune deficiency patients such as hematological tumors, and the proportion of mixed infections is high. mNGS has the ability to detect multiple pathogens at the same time, which also has advantages.

Among the patients included in this study, 202 had hematologic malignancies, and 58 were admitted to the ICU, all with severely compromised immune function, making blood mNGS testing a crucial diagnostic method in this patient population where invasive sampling may not be feasible.

In our daily living environment, the concentration of *Aspergillus* spores typically ranges from 1 to 100 spores/m^3^, reaching up to 10^8^ spores/m^3^ in certain specific environments (Tegelaar and Wösten, 2017). Therefore, isolating *Aspergillus* species from respiratory cultures of asymptomatic individuals without evidence of invasive or allergic disease is quite common (Mortensen et al., 2011; Máiz et al., 2015; Nguyen et al., 2015). The proportion of *Aspergillus* DNA found in lung biopsy specimens from healthy adults is reported to be 37% (Denning et al., 2011). This study observed a false-positive rate of 40.1% in the mNGS detection of *Aspergillus*, which may be attributed to environmental contamination during sampling, transportation, and the testing process.

Patients with neutropenia are prone to invasive aspergillosis. In this study, both the infection and contamination groups primarily had underlying hematologic diseases, resulting in varying degrees of neutropenia. However, there was no statistically significant difference in white blood cell count and neutrophil count between the infection and contamination groups.

This study has several limitations. Firstly, being a single-center study, the enrolled patients were predominantly characterized by hematologic diseases, and the site of *Aspergillus* infection was mostly limited to the lungs (98%), indicating a lack of diversity. Secondly, as a retrospective study, it could not evaluate the sensitivity and specificity of mNGS in diagnosing *Aspergillus* infection Thirdly, the study included only two confirmed cases, while there were 67 possible cases, which may introduce bias in assessing the diagnostic efficacy of mNGS for *Aspergillus* infection. Future research should focus on prospective, multicenter studies with a larger sample size to further evaluate the efficacy of peripheral blood mNGS in diagnosing invasive aspergillosis.

## 5 Conclusion

Peripheral blood mNGS exhibits a high positive predictive value in the diagnosis of invasive aspergillosis. It can be employed in critically ill patients, aiding in the earlier diagnosis and initiation of treatment when combined with high-risk factors and clinical characteristics.

## Data Availability

The original contributions presented in the study are publicly available. The mNGS datasets have been submitted to NCBI Sequence Read Archive (SRA) under the Bioproject accession number PRJNA1140277.
