# Supplementary material for: Value of plasma metagenomic next-generation sequencing for the diagnosis of invasive aspergillosis: a multicenter-center retrospective study
Source: Front Cell Infect Microbiol. 2025 Nov 21;15:1656233. doi: 10.3389/fcimb.2025.1656233 (PMC12679094; doi:10.3389/fcimb.2025.1656233)
Supplement: Supplementary file 1 [file DataSheet1.pdf]

Supplementary

Plasma mNGS testing

1. Sampling

Peripheral venous blood samples were collected in Geneseek serum cell-free DNA tubes (GS-CF-05). Generally, 10ml peripheral venous blood (2 tubes) is required to be drawn from from each patient. The collected specimens need to be stored at room temperature and sent to the clinical laboratory as soon as possible. After receiving the specimens, the clinical laboratory immediately separates the plasma under the centrifugal parameters of 500 g for 5 min at 4℃. The separated plasma is used to extract cell free DNA (cfDNA) and cell free RNA (cfRNA).

2. Nucleic Acid Extraction, Library Preparation, and Sequencing

cfDNA was extracted from 200 µL plasma using a QIAamp® Circulating Nucleic Acid Kit (Qiagen, Cat No./ID: 55114) following the manufacturer's protocol. CfRNA was extracted with a QIAamp® Viral RNA Kit (Qiagen, Cat No./ID: 52904). DNase was used to treat crRNA. cDNA was generated using reverse transcriptase and dNTPs (Thermo Fisher). Libraries were constructed for the DNA and cDNA samples using a Nextera XT DNA Library Prep Kit (Illumina, San Diego, CA). Index PCR parameters were as follows: 68°C for 3 min and 98°C for 3 min, followed by 18 cycles of 45 s at 98°C, 30 s at 62°C, and 2 min at 68°C, before a final minute at 68°C. Dual indexing was conducted by employing the IDT for Illumina DNA/RNA UD indexes (catalog number 20027213). The size 1 distribution was measured on the Agilent 2100 Bioanalyzer using the High Sensitivity DNA Kit (Agilent, Eugen, USA) and the concentration of the libraries was quantified by the Qubit dsDNA HS Assay kit on a Qubit 3.0 flurometer (Thermo Fisher Scientific, Waltham, MA, USA). Library pools were then loaded onto the Illumina Nextseq CN500 sequencer for 50 cycles of single-end sequencing (SE-50), generating approximately 20 million reads for each library.

3 Bioinformatics Analyses

Low-quality reads, adaptor sequences, repeated reads, as well as those shorter than 50bp in the raw data are removed with Trimmomatic. Low complexity reads were filtered by Kcomplexity using default parameters. Human sequence data were identified and excluded by mapping to a human reference genome (GRCh38) using SNAP v1.0beta.18. In order to construct the microbial genome database, pathogens and their genomes or assemblies were selected following the Kraken2 criteria for selecting representative assemblies for microorganisms (bacteria, viruses, fungi, protozoa, and other multicellular eukaryotic pathogens) from an open access kraken2 database

(https://benlangmead.github.io/aws-indexes/k2). Pathogens from Johns Hopkins ABX Guide (https://www.hopkinsguides.com/hopkins/index/Johns_Hopkins_ABX_Guide/Pathogens), and clinical case reports or research articles published in current peer-reviewed journals are included in this in-house database (see in supplementary table S1). Microbial reads were aligned to the database using Burrows-Wheeler Aligner software.

We defined that reads with less than 4 mismatches were mapped reads. Reads with multiple locus alignments within the same genus were excluded in secondary analysis. Only reads mapped to the genome within the same species were considered.

4. Quality Control

We designed a run-specific negative control (NC) sample and two positive control samples to monitor false negatives and false positives that may occur during the experiment. The NC sample refers to sterile deionized water added with 105 peripheral blood mononuclear cells (PBMC) from healthy donors. The two positive control samples were prepared by adding approximately 103 copies of Nocardia farcinica (a thick-walled fungus) and 103 copies of Influenza B virus (an RNA virus) to the negative control samples, respectively. All the control samples are tested in parallel with the clinical samples from the beginning of sample processing to bioinformatics analysis.

5. Reporting

The reporting team in our laboratory consists of 6 persons whose specialties involve pathogen molecular diagnosis(n=1), clinical microbiology and infection(n=2), infection immunology(n=1), and clinical virology(n=1) and infectious disease epidemiology(n=1). The reporting team selects organisms to report to the treating clinical team based on a technical threshold and a clinical prediction rule. The technical threshold (to determine whether an organism is detected).

a. for a given species also detected in the NC sample, we determined whether the species was detected by calculating a SMRN ratio (SMRN-r). The SMRN-r was defined as the SMRNsample / SMRNNC (i.e., the SMRN corresponding to a given species in the clinical sample divided by the SMRN in the NC sample). if the SMRN-r ⩾10, the species is considered to be detected.

b. for a given species which was not detected in NC sample will be considered to be detected when SMRN was no less than 3. (Special situation: If some species that are highly related to the clinical manifestations of the patient, even if the SMRN is less than 3, it is considered to be detected. However, this should be decided through a detailed medical history review and discussion with the treating physician.)

c. for an important human pathogen that was parasitic in cells and difficult to extract nucleic acid (e.g., Mycobacterium spp., Nocardia spp., Brucella spp., etc.), was considered

as positive when SMRN >1.

d. the SMRN of a DNA/RNA virus should cover three or more non-overlapping regions on the reference genome to be considered detected.

The laboratory analytical rules (to determine whether an organism should be reported) Under the premise of meeting the above technical threshold, it is necessary to further distinguish between potentially pathogenic and non-pathogenic organisms. In our laboratory, an organism will be considered as a potentially pathogen and report to the treating clinicians if one of the following rules were met:

a. the organism also detected by other microbiological tests (culture, serology, and/or PCR) performed within seven days of presentation.

b. for samples with multiple organisms detected, report the organisms with a relative abundance of SMRN greater than 30% at the species level in the same taxonomy (bacteria, fungus, parasite, virus).

c. If an organism is highly suspected to be related to the clinical manifestations of the patient after reviewing the electronic medical record or discussing with the clinicians, or the evidence of the organism causing the disease have been reported in literature or books, it should also be reported.

Supplementary Table. Details of identifying different genus of *Aspergillus* using different methods

	Culture of tissue	Culture of sputum or BALF	Plasma mNGS	BALF mNGS	Sputum mNGS	Cerebrospinal fluid mNGS
A. fumigatus (n=102)	2	22	102	4		
A. flavus (n=132)		8	132	8	6	2
A. terreus (n=6)			6			
A. oryzae (n=30)			30	2		
A. niger (n=34)		4	34			
A. clavatus (n=2)			2			1
A. ustus (n=2)		2	2			
A. tamarii (n=10)			10			
A. nomius (n=4)			4			
A. carbonarius (n=2)			2			
A. chevalieri (n=8)			8			
